# Brain death determination in Europe: one condition with too many nuances

**DOI:** 10.1186/cc13654

**Published:** 2014-03-17

**Authors:** IA Crippa, A Bronco, A Vargiolu, G Citerio

**Affiliations:** 1 San Gerardo Hospital, Monza, Italy

## Introduction

A patient is declared brain dead (BD) when physicians determine permanent loss of brain functions. Unfortunately, criteria for defining BD vary across different countries [1]. We therefore decide to survey BD diagnostic modalities in Europe in order to describe differences.

## Methods

A multiple-choice questionnaire was developed on an online platform [2]. Direct link was sent to national representatives of the European Society of Intensive Care Medicine and NeuroIntensive Care section's members. Thirty-three countries were contacted. Answers were reviewed. In cases of discrepancies or missing data, participants were contacted for further clarification. Descriptive statistics have been applied.

## Results

Twenty-eight participants returned the questionnaire (85%). Every country has either specific law (93%) or guidelines issued by the scientific society (89%). Clinical examination, essential to the diagnosis, is the only requirement in 50% of countries. Coma, apnea, absence of corneal and cough reflexes are always necessary. Blood pressure and electrolytes are checked in 64% as mandatory prerequisites. The apnea test is legally defined in 86% of countries. Eighty-two percent of countries require achievement of a target paCO_2 _level while the Netherlands' law states target apnea duration. Number of physicians (median 2, range 1 to 4), number of clinical examinations (median 2, 1 to 3), and minimum observation time (median 3 hours, 0 to 12) are variable requisites in different countries. In 50% of nations, additional tests are required. Hypothermia (4%), anoxic injury (7%), inability to complete clinical examination (61%), toxic drug levels (57%), and inconclusive apnea test (54%) are legal indications to perform additional tests. Cerebral blood flow investigation is mandatory in 18% of countries, while it is either optional or used only in selected cases in 82%. Conventional angiography is still the preferred method (50%), followed by transcranial Doppler (43%), angioCT (39%), CT perfusion and angioMR (11%). EEG is always (21%) or optionally (14%) recorded. Russia and Croatia evaluate both EEG and cerebral blood flow (7%).

## Conclusion

There are still areas of uncertainty and disparities in brain death diagnosis in European countries. This predisposes to misdiagnosis and confusion both for clinicians and families. Measures to promote uniformity of brain death procedures and clinical practice are therefore desirable.
